# Impact of Supplementary Feeding on Reproductive Success of White Storks

**DOI:** 10.1371/journal.pone.0104276

**Published:** 2014-08-13

**Authors:** Roland Hilgartner, Daniel Stahl, Dietmar Zinner

**Affiliations:** 1 Affenberg Salem, Salem, Germany; 2 Department of Biostatistics, King's College London, Institute of Psychiatry, London, United Kingdom; 3 Cognitive Ethology Laboratory, German Primate Center, Göttingen, Germany; Phillip Island Nature Parks, Australia

## Abstract

European white stork (*Ciconia ciconia*) populations have been object to several conservation measures such as reintroduction programs, habitat improvement or supplementary feeding in the last decades. Although recent white stork censuses revealed an upward trend of most of the western populations, evaluations of the relative importance of certain conservation measures are still scarce or even lacking. In our study we analyzed the effect of supplementary feeding on the reproductive success of white storks in conjunction with other factors such as weather or nest site characteristics. We present data of 569 breeding events at 80 different nest sites located in variable distances to an artificial feeding site at Affenberg Salem (south-western Germany) collected from 1990–2012. A multilevel Poisson regression revealed that in our study population (1) reproductive success was negatively affected by monthly precipitation in April, May and June, (2) pairs breeding on power poles had a lower reproductive success than pairs breeding on platforms or trees and (3) reproductive success was significantly higher in pairs breeding in close distance to the feeding site. The number of fledglings per nest decreased by 8% per kilometer distance to the feeding site. Our data suggest that supplementary feeding increases fledgling populations which may be a tool to attenuate population losses caused by factors such as habitat deterioration or unfavorable conditions in wintering habitats.

## Introduction

White stork (*Ciconia ciconia*) populations in Western Europe decreased tremendously after 1945 leading to a severe threat of extinction or even extinction in most West European countries [1]. Breeding white storks disappeared in Belgium, Switzerland (1950) and in Sweden (1955) [2], [3]. A strong decline of the population was observed in the Netherlands (down to 5 pairs 1984), Denmark (down to 6 pairs in 1996) and France (down to 11 pairs 1974) [4]–[6]. Environmental changes in breeding and wintering habitats have been discussed as main reasons for the severe decline of West European populations [7]–[11]. As a consequence conservation actions on a national level such as reintroduction projects, supplementary feeding and habitat improvement have been applied in several West European countries to prevent migrating white storks from extinction [8], [12]. Reintroduction activities included continuous releases of white storks reared in captivity, installation of nest sites (poles) and supplementary feeding of free flying individuals [12].

White stork censuses in 1995 and 2004 revealed an upward trend of most western populations [9], [13]. Causes for the recovery of western white stork populations are discussed multidimensional. Reintroduction of white storks reared in captivity and less severe droughts in West African wintering habitats are regarded as key aspects for the recovery of certain populations [9], . Additionally, alternative food resources in wintering habitats such as rubbish dumps or the copious availability of invasive species (e.g., red swamp or Louisiana crayfish, *Procambarus clarkii*) are discussed to influence population recovery of white storks [9], [15], [16]. However, some of these management efforts have been stopped because conservation programs are often limited in space and time. Yet, analyses to what degree different conservation actions such as supplementary feeding, habitat improvement and reintroduction contributed to the recovery of West European white stork population are still scarce (but see [17], [18], [19]) and, to our knowledge, the effect of additional feeding on reproductive success of white storks has never been investigated in detail.

In most of the west European white stork populations reproductive success is low or highly variable [1], [20]–[22]. Multiple factors may affect the number of fledglings, including age, arrival date, breeding experience of pairs and physical fitness of parent storks [19], [23], [24], weather condition during the nestling period [22], [25], [26], nest site characteristics [27] and food availability [10], [11], [19], [26], [28], which depends largely on habitat quality. Here certain human farming practices can have a major negative impact [29]–[31]. Food availability is considered as a limiting factor for breeding success and hence for population trends [10], [11], [19], [26], [28]. Supplementary feeding of free flying white storks as a conservation measure should therefore compensate for low habitat quality and increase reproductive success of pairs [19], [32], [33].

Supplementary feeding is a common element of wildlife management that can aid in the establishment of a viable, self-sustaining population by increasing body condition, growth rates, survival, social interactions or reproductive success (e.g. [34]–[38], but for possible negative effects see also [31], [39]). However, the effects of supplementary feeding on reproduction rates and life history of white storks have never been evaluated in detail. The aim of this study was to explore effects of supplementary feeding in conjunction with a number of other factors proposed to affect reproductive success in white storks such as weather [22] and nest site characteristics [27]. We present data collected over a period of 23 years on reproductive rates of free flying pairs of white storks breeding in variable distances to a supplementary feeding site at Affenberg Salem (Baden Württemberg, Germany).

White storks, while breeding, forage on average in an area of not more than 1500 m from the nest site [40] and Massemin-Challet et al. 2006 [19] showed that pairs breeding close to a rubbish dump (<1000 m) fledged more young than pairs breeding further away. We therefore predicted that storks, nesting closer to the supplementary feeding site at the Affenberg had a higher reproductive success, measured as number of fledglings per nest and year, than storks nesting in greater distances.

## Methods

### Study Area

**Figure 1:**
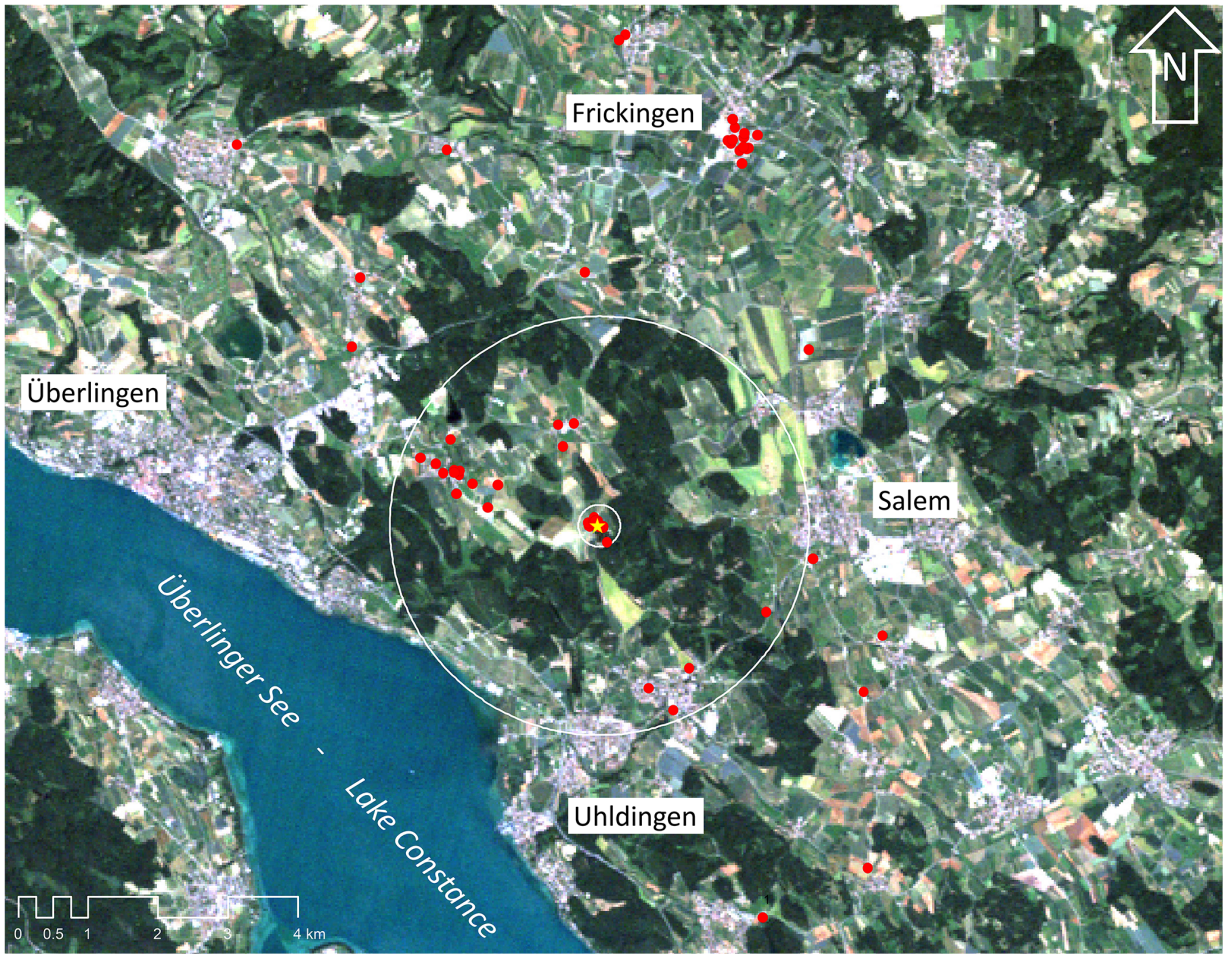
Geographic position of white stork nest sites (red dots) in the vicinity of the Affenberg, Salem. The yellow star indicates the artificial feeding site and the white lines encircle the feeding site with a distance of 0.3 km and 3.0 km, respectively (background image derives from Landsat 4–5 TM scene, path/row 194/27, 08.07.2010; http://glovis.usgs.gov/).

The study was conducted at Affenberg Salem (N 47°45′43″ E 09°14′44″) Baden-Württemberg, Germany (Fig. 1). Affenberg Salem harbours one of the largest colonies of free flying white storks belonging to the western population in the southern part of Germany. The colony was founded at Affenberg Salem with nine reintroduced individuals in 1978. In the last 34 years, the colony attracted wild breeding storks, reaching a current size (2012) of 28 breeding pairs located directly at Affenberg Salem and 36 pairs breeding in the surroundings. Nest sites are located at variable distances (min distance 21 m, max distance 7527 m; Fig. 1) to the feeding site at Affenberg Salem and all breeding sites are encircled by a mixture of intensively cultivated fields, plantations, meadows and ponds. Within the study period (1990–2012) the colony consisted exclusively of free flying pairs.

### Food provisioning at Affenberg Salem

At a defined feeding site at Affenberg Salem, supplementary food (small fish and one-day old chicken) was provided twice a day (11.00 h and 16.45 h) during the breeding season (including the nestling period) and once a day (14.00 h) outside the breeding season. The food was distributed randomly over an area of approximately 1000 m^2^. The number of storks visiting the feeding site was highly variable depending on season (Hilgartner, unpublished data) and most likely also on food availability in the surroundings. During the breeding season number of storks visiting the feeding site was highest but at times dropped to 0. Outside the breeding season number of storks at the feeding site was in general considerably lower. The majority of the population is migrating and therefore not present during winter feeding. We tried to adjust the quantity of food so that 30 min after the food had been distributed no more food was left at the feeding site. As an estimate for adjustment, we used the time storks needed to empty the feeding site the respective day before. As consequence feeding time per day and the amount of food provisioned were limited. The amount ranged between (0–35 kg of chicken and 0–10 kg of small fish per feeding session). Storks were able to ingest up to 12 chickens per feeding into their crops and transport them to their nests (pers. obs. D. Zinner).

### Data Collection

Breeding pairs were determined following standardized census methodology [9]. Breeding activity of all pairs was monitored regularly. If possible pairs were identified via metal or PVC rings. We don't have identities of all breeding pairs because not all individuals had rings and in some years/cases it was not possible to decipher the rings with certainty. Therefore, data on individual fitness of breeding storks, their ages or pair experience are not available. Data on clutch size, hatching success and chick mortality were collected *ad libitum* and were only available for a few pairs. As a measure for reproductive success we used number of fledged chicks per breeding pair (nest) per year. As potential predictor variables we included (1) distance between nest site and feeding site (km, determined in ArcGIS 10, ESRI Inc.), assuming that storks nesting closer to the feeding site will obtain supplementary food more regularly and in larger quantities, (2) weather data for the breeding and nestling periods, i.e., monthly rainfall and average temperature for April, May and June 1990–2012, obtained from the closest meteorological station of the Deutscher Wetterdienst in Konstanz, which is about 10 km from the feeding site and in addition (3) type of nest base: (a) on artificial platforms, (b) wild nests on houses and in trees (not particularly supported nests) or (c) on power poles. Data are available from Table S1.

### Statistical Analysis

We used a multilevel Poisson regression to examine potential associations between the number of fledglings and the distance to the feeding site while controlling for other potential predictors variables. As covariates we assessed monthly rainfall and average temperature in April, May and June of respective years and nest base type. We included nest ID as a random factor to model the dependency of breeding success due to the same nest (as a proxy for intrinsic nest-site quality, individual breeding stork quality, breeding pair tenure or nest-site fidelity of parent storks, which were in fact not known in most cases).

**Figure 2:**
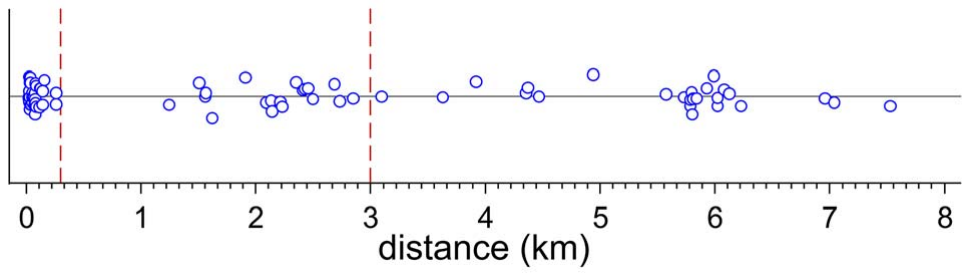
Dotplot of distances between white stork nest sites and feeding site (used to determine cut-off points). Each dot represents one nest site. Data points are slightly jittered (adding random noise to data) in order to prevent overplotting of data points. Vertical lines indicate cut-off distances.

In a first step, we included all independent variables in the model and subsequently removed variables with a p>0.1. We then compared this final model with (1) a model with replacing weather data with ‘year’ as a categorical independent variable (‘good stork years’ vs. ‘bad stork years’) and (2) a model with distance replaced by a categorical variable with either two (<300 m or >300 m) or three levels (<300 m, 300 m–3 km, >3 km) (Fig. 2). The three models are not nested and cannot be assessed by statistical tests. Therefore we compared the models using AIC [41].

A Poisson model assumes that the variance is equal to the mean. If there is more variation in the number of fledglings than predicted by the model (‘overdispersion’, e.g. due to more zero observations – in our case: no fledglings) standard errors are often underestimated and therefore p-values (and confidence intervals) become overoptimistic (type I error inflation). To account for possible overdispersion we used an observation–level random effect to model possible overdispersion assuming that overdispersion arises from errors taking on a log-normal mixing distribution [42], . This method also allows estimating and comparing the contribution of explained variance of the different variables [43]. We calculated the increase in the amount of variance in the response explained by adding a variable of interest, given that all other fixed and random variables are in the model (unique explained variance) using the approach described by Nakagawa and Schielzeth 2013 [44] for generalized linear mixed effects models. Incidence risk ratio (IRR) and 95% confidence intervals (95% CIs) of the final models are presented.

### Ethical statement

Besides handling of white stork chicks for ring application, no other handling of storks was done. For all nests included in this study ring application was done by one of the authors (RH) and Walter Angst who hold a ringing permit issued by Regierungspräsidium Tübingen and Landratsamt Bodenseekreis (Germany). All handling of the storks was done in compliance with the laws of Baden-Württemberg (Germany). Number of fledglings was determined from a distance, if necessary with the help of binoculars. For these observational data collection no specific permission was required.

## Results

Over a period of 23 consecutive years we collected data from 569 breeding events at 80 nest sites. The minimum number of nests per year was 10 in 1991. This number increased to 64 in 2012 (mean number of nests per year  = 24.7, SD = 14.6). The number of fledglings per brood ranged from 0 to 5 (mean  = 2.2, SD = 1.6, n = 569). ‘Bad years’ for stork reproduction were 1991 (0.9±1.3 fledglings per nest [mean ± SD, n = 10]) and 2007 (0.9±1.03 fledglings per nest [mean ± SD, n = 35]). In contrast, ‘good years’ were 2003 (3.0±1.6 fledglings per nest [mean ± SD, n = 22]) and 2011 (2.9±1.10 fledglings per nest [mean ± SD, n = 50]). On average 2.46 (SD = 1.53, N = 288) young storks fledged if breeding occurred in close proximity to the feeding site (<300 m); 1.99 (SD = 1.62, N = 148) chicks fledged in nests which were between 300 m and 3 km away form a feeding site and only 1.70 (SD = 1.45, N = 133) chicks fledged from nests if the distance to the feeding site was more than 3 km.

**Figure 3:**
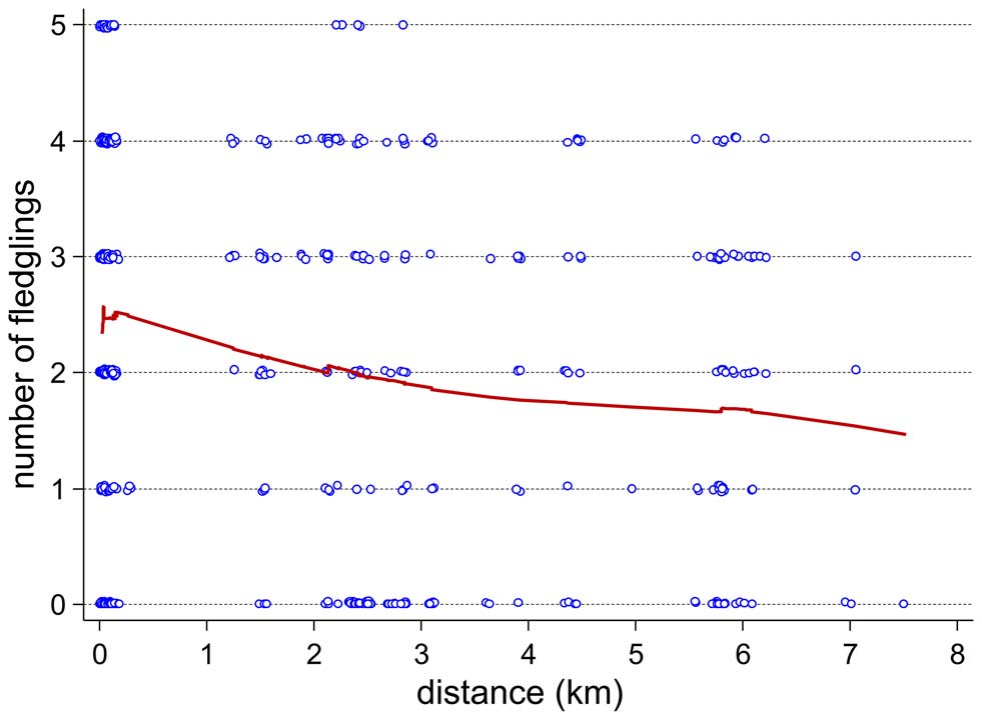
Number of fledglings per nest and year in relation to distance between the feeding place and nest site (km). A Lowess (locally weighted scatterplot smoothing) curve is used to describe the trend of the data. Data points are slightly jittered (adding random noise to data) in order to prevent overplotting of data points.

Our overall final model explained 28.7% of the variance. The fixed effects ‘nest base’, ‘distance to feeding site’ and ‘monthly precipitation in April, May and June’ explained 15.5% and the random factor ‘nest ID’ 13.2% (Table 1). Monthly mean temperatures did not predict significantly mean number of fledglings and were not part of the final model (all p>0.18) (Table 1). A model with year as a categorical variable instead of weather data resulted in a poor model (delta AIC compared to final model  = −17.9). The final model suggests a negative relation between distance to feeding site and number of fledglings per nest and year (Fig. 3) with a reduction of the number of fledglings by 8% per kilometer distance from the feeding site (Incidence rate ratio  = 0.92, unique r^2^ = 0.045).

**Table 1:** Results of multilevel Poisson regression with nest ID as a random factor.

Variable	Incidence rate ratio (95% C.I.)	chi^2^	z	p
nest base		5.56		0.06
power pole vs platform	0.69 (0.51–0.94)		−2.35	0.019
wild vs. platform	0.98 (0.75–1.29)		−0.15	0.88
power pole vs. wild	0.708 (0.484–1.036)		1.78	0.075
distance (km)	0.920 (0.88–0.96)		−3.60	<0.001
preci April (mm)	0.997 (0.995–0.999)		−3.46	<0.001
preci May (mm)	0.996 (0.994–0.997)		−5.42	<0.0001
preci June (mm)	0.997 (0.995–0.999)		−3.19	<0.001

Rate ratios (95% confidence intervals) are shown with chi^2^ or z test statistics and p values. An observation–level random effect was included to model possible overdispersion. Variance of i) random effects “nest ID”: 0.076 (95% C.I.: 0.035–0.17), p<0.05 and ii) observation-level random effect: 0 (exact 1.86e-23), n.s. The overall model explained 28.7% of the variance of which the random factor nest ID explained 13.2% and the fixed effects nest base, distance and precipitation in April, May and June 15.5%.

In addition, monthly precipitation in April, May and June had an independent negative effect on the number of fledglings (combined unique r^2^ = 0.075). The type of nest-base tends to have an influence with nests on power poles having 31% less fledglings than storks breeding on platforms and 29% less fledglings than storks breeding in wild nests, whereas nests on platforms and wild nests on trees and buildings yielded similar numbers of fledglings (unique r^2^ for factor nest base  = 0.02, Table 1).

Using distance as a categorical variable with two (<300 m or >300 m) and three levels (<300 m, 300 m–3 km, >3 km) resulted in a model with a slightly smaller AIC (2 levels: delta AIC  = −0.77, 3 levels: delta AIC  = −2.77) than the model with distance as a continuous variable. The 2-levels model suggests that storks breeding in a distance larger than 300 m from the feeding site have 29% less fledglings (IRR: 0.71 [95% C.I.: 0.585–0.86], z = −3.4, p = 0.001, unique r^2^ = 0.048) than storks breeding in a distance of less than 300 m from the feeding site. Using distance as a variable with three levels suggests that storks breeding in a distance of more than 3 km from the feeding site have 36% less fledglings (IRR: 0.64 [95% C.I. 0.49–0.82], z = −3.48, p<0.0001) than those breeding close to the feeding site (<300 m) while storks breeding in an intermediate distance (300 m–3000 m) have 20% less fledglings. But this difference is not significant compared to those living less than 300 m to the feeding site (IRR: 0.80 [95% C.I. 0.62–1.02], z = −1.79, p = 0.075). There is also no significant difference in the number of fledglings between those breeding in an intermediate and a long distance to the feeding site (IRR: 0.80 [95%C.I. 0.61–1.04], z = −1.64, p = 0.10). The distance factor accounted for 4.6% of the explained variance.

## Discussion

Our study provides a first step to understand the effects of a particular conservation measure in a West European white stork population and to evaluate its consequences. We analyzed the effect of supplementary feeding on the reproductive success of white storks, which is a key variable in determining population trends [10], [11], [26], [28], [45], [46]. We included additional variables considered to influence reproductive success such as mean monthly temperature, monthly precipitation and type of nesting platforms and as a random factor ‘nest ID’ in our analyses.

The random factor ‘nest ID’ explains a large amount of the variance. Since we were not able to determine the identities of the breeding storks in all cases with certainty and the habitat quality surrounding the nests, we used this factor as a proxy for individual breeding stork quality and pair tenure, but also for quality of nest and the habitat around the nest, factors which are expected to influence reproductive success of breeding pairs [11], [21], [47]. It is therefore not surprising, that ‘nest ID’ has a significant impact on fledgling success in our study population.

Furthermore, environmental stressors such as extreme weather conditions affect survival rates in white storks [22] and indeed, breeding success was negatively affected by monthly precipitation in April, May and June but not by mean monthly temperatures. In our study population, most birds hatched in April and May with few outliers in March and June [Hilgartner & Angst unpublished data]. In the first three weeks after hatching, chicks do not have thermoregulatory abilities [48]. Even between three and five weeks after hatching, thermoregulation is still affected when chicks are too big to be protected by their parents but still have not fully developed their plumage [40], [49]. In accordance with our study Carrascal *et al*. [50] did not find an effect of mean spring temperatures on reproductive success, but number of rainy days was decisive for chick survival, with higher number of rainy days leading to lower chick survival. A number of other studies have also found a negative impact of rainy springs on the breeding success of white storks which seems to be general pattern across different populations [22], [26], [40].

The type of nest base tends to have an influence on reproductive success of white storks in our study population. White storks using nests of power poles had a lower number of fledglings than pairs breeding on platforms or wild nests. This effect is in contrast to the study of Tryjanowsky *et al*. [27] where no difference was found between the reproductive success of white storks nesting on power poles and other nesting sites such as chimneys, trees or roofs. A possible explanation for this difference maybe the yearly nest cleaning in autumn performed in our study colony. Nest cleaning involves the removal of the uppermost layer and the removal of rubbish e.g. strings, plastic etc. Nests on power poles, however, were excluded from cleaning as these nests are not easily accessible without presence of electricity suppliers and shutting off electricity. The cleaning may have an influence on survival of chicks as rubbish can have fatal effects on nestlings when they swallow plastic or get entangled in strings ([51]; Hilgartner pers. obs.). Moreover, nest cleaning may also lead to a better water permeability of the nest which could be advantageous for chick survival during long-lasting periods of rain.

Reproductive success of breeding white storks was significantly higher in nests in close distance to the artificial feeding site, as the number of fledglings decreased by 8% per kilometer distance from the feeding site. A similar observation was reported by Massemin-Challet et al. [19] who found that storks nesting near (<1000 m) a reliable artificial food source in Alsace produced significantly more fledglings than storks breeding further away. A plausible explanation for this negative correlation is that storks breeding closer to the feeding site made more frequently use of it and were able to supply their chicks with more food, which resulted in a higher reproduction. During breeding, white storks usually forage in close distance to their nest sites [20], [40], [47], [52], [53]. Moritzi et al. [40] found that 88% of all foraging records were within a radius of 1 km with a median distance of 380 m to the breeding colony. Hence, for white stork pairs not breeding in close distance to the feeding site greater flight expenditure might not compensate for supplementary food. Moreover, especially during the first weeks after hatching, at least one pair partner stays at the nest all the time for guarding the nestlings [54], [55]. Feeding times and food availability at the feeding site is limited. As a consequence pairs breeding in close distance to the feeding site are able to perform more successful provisioning flights than pairs of more distant nests. Hence, breeding storks in the population do not equally profit from supplementary feeding most probably leading to the observed distance related reproductive success in our stork colony.

A general impact of food availability on reproductive success in white storks has been found in a study by Tryanowski & Kuźniak [28]. They showed that reproductive success of white storks was higher in years with high food availability. A similar effect was found in pairs breeding in close distance to optimal feeding habitats such as meadows, pastures and wetlands [11], [52], [56]. Pairs breeding in close distance to such optimal feeding sites had a higher reproductive success than pairs breeding in suboptimal habitats. The intake of additional high quality food might increase the physical fitness of parent storks, in particular that of females and might therefore increase clutch size, egg size or investment in feeding rates of chicks [28], [53]. Unfortunately, we do not have data on these factors for our study population. However, it seems most likely that the main effect responsible for successful fledging is food availability for chicks during the nestling period [19], [21], [26], [57], [58].

Although supplementary feeding as a management tool has been controversially discussed [32], [33], [59] a raising number of studies underline its conservation relevance for several species (e.g., Florida shrub jay, *Aphelocoma coerulescens*, [38]; vultures, [60], [61]; Spanish imperial eagle, *Aquila adalberti*, [62], brown teal, *Anas chloris*, [63]; Iberian lynx, *Lynx pardinus*, [64]). Our study demonstrates that supplementary feeding enhances reproductive success, measured as number of fledglings, of white storks breeding in close proximity to the feeding site.

In West European White Storks overall reproductive success is still low [17], [21], [22], [40] and comparable to the low reproductive success during the dramatic population decline. Hence carrying capacity of habitats in many regions may not be sufficient to sustain a viable population. This could be also the case for our study population given the low reproductive success of 1.7 fledglings at the periphery compared to 2.5 fledglings in close distance to the feeding site (<300 m).

**Figure 4:**
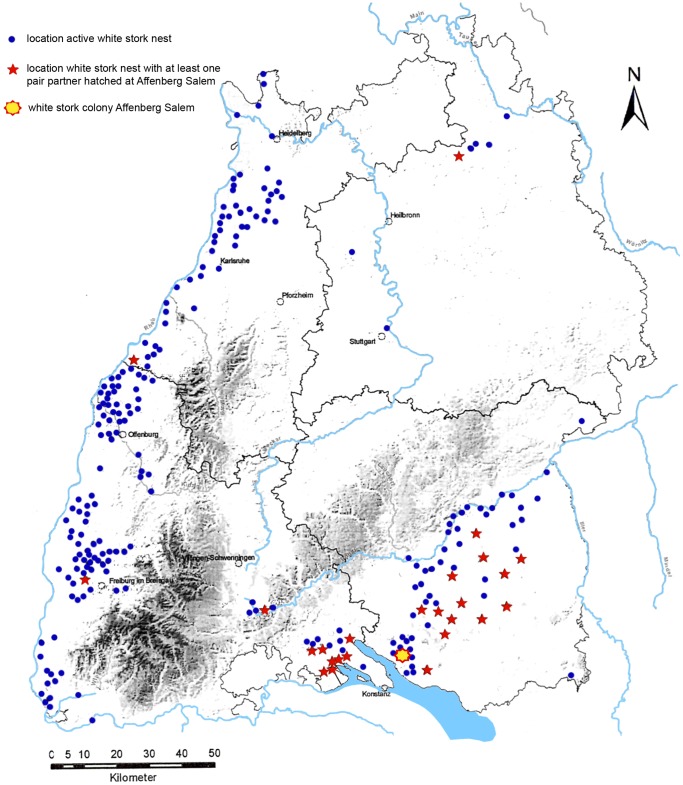
Location of active white stork nests in south-western Germany in 2007 (dots and stars). Nest locations with at least one breeding partner hatched in the Affenberg colony are indicated with stars. (Walther Feld; Storchenbeauftragter Baden-Württemberg unpublished data).

The increased fledgling population in close distance to the feeding site may have contributed to a positive population effect, at least in our study population. Storks fledged at Affenberg Salem recolonized several regions of Baden-Württemberg (south-western Germany, Fig 4) and ringing data indicate that about 34% of the 2012 breeding population in Salem fledged at the Affenberg colony (less than 300 m from the feeding site; Hilgartner unpublished data). However, to fully understand to which extent additional feeding, applied as a conservation tool in several parts of Baden-Württemberg and other regions of Western Europe has contributed to the population recovery of West European white storks, it is necessary to study the origin of the actual breeding population and the origin of the recruits globally. Furthermore, effects of additional feeding have to be evaluated in relation to other conservation measures, such as reintroduction programs or habitat restoration. Only if we assess the impact of various conservation measurements and their interactions we will be able to make sound conservation decisions. This is of special significance in a species that still has to cope with dramatic changes in its breeding and wintering habitats and that still suffers from low overall reproductive success.

## Supporting Information

Table S1 (XLSX)
